# Morphological Features and Important Parameters of Large Optic Discs for Diagnosing Glaucoma

**DOI:** 10.1371/journal.pone.0118920

**Published:** 2015-03-23

**Authors:** Satoshi Okimoto, Keiko Yamashita, Tetsuo Shibata, Yoshiaki Kiuchi

**Affiliations:** 1 Department of Ophthalmology and Visual science, Graduate School of Biomedical Sciences, Hiroshima University, Hiroshima, Japan; 2 Shibata Eye Clinic, Hyogo, Japan; Bascom Palmer Eye Institute, University of Miami School of Medicine;, UNITED STATES

## Abstract

**Purpose:**

To compare the optic disc parameters of glaucomatous eyes to those of non-glaucomatous eyes with large discs.

**Methods:**

We studied 225 consecutive eyes with large optic discs (>2.82 mm2): 91 eyes with glaucoma and 134 eyes without glaucoma. An eye was diagnosed with glaucoma when visual field defects were detected by the Humphrey Field Analyzer. All of the Heidelberg Retina Tomograph II (HRT II) parameters were compared between the non-glaucomatous and glaucomatous eyes. A logistic regression analysis of the HRT II parameters was used to establish a new formula for diagnosing glaucoma, and the sensitivity and specificity of the Moorfields Regression Analysis (MRA) was compared to the findings made by our analyses.

**Results:**

The mean disc area was 3.44±0.50 mm2 in the non-glaucomatous group and 3.40±0.52 mm2 in the glaucoma group. The cup area, cup volume, cup-to-disc area ratio, linear cup/disc ratio, mean cup depth, and the maximum cup depth were significantly larger in glaucomatous eyes than in the non-glaucomatous eyes. The rim area, rim volume, cup shape measurement, mean retinal nerve fiber layer (RNFL) thickness, and RFNL cross-sectional area were significantly smaller in glaucomatous eyes than in non-glaucomatous eyes. The cup-to-disc area ratio, the height variation contour (HVC), and the RNFL cross-sectional area were important parameters for diagnosing the early stage glaucoma, and the cup-to-disc area ratio and cup volume were useful for diagnosing advanced stage glaucoma in eyes with a large optic disc. The new formula had higher sensitivity and specificity for diagnosing glaucoma than MRA.

**Conclusions:**

The cup-to-disc area ratio, HVC, RNFL cross-sectional area, and cup volume were important parameters for diagnosing glaucoma in eyes with a large optic disc. The important disc parameters to diagnose glaucoma depend on the stage of glaucoma in patients with large discs.

## Introduction

Glaucoma is a progressive optic neuropathy in which morphological changes of the optic disc and the retinal nerve fiber layer (RNFL) progress to visual field defects [1,2]. Because the rim area of the optic disc is reduced in glaucomatous eyes, evaluating the changes of the optic disc morphology is essential for diagnosing glaucoma [3–7]. In general, the optic disc cup area is increased in glaucomatous eyes, and the cup-to-disc ratio is used for diagnosing glaucoma [8]. However, the cup area is also large in eyes with a large optic disc, and these eyes may not be glaucomatous [9,10]. Therefore, a careful examination is needed to discriminate whether a large cup area is due to glaucoma or to a large optic disc.

The Heidelberg Retina Tomograph II (HRT II; Heidelberg Engineering, GmbH, Heidelberg, Germany) can obtain highly reproducible measurements of the stereometric parameters of the optic disc, and these parameters can be used to differentiate normal from glaucomatous eyes. Therefore, the HRT II has been used for glaucoma screening [11–13].

The Moorfields Regression Analysis (MRA) of the stereometric parameters obtained by the HRT II is a useful method of diagnosing glaucoma based on the morphological parameters of optic discs. The MRA program is based on comparing the rim area and disc area of a glaucoma suspect to the normal database embedded in the instrument [11]. From the results of the comparisons, a determination can be made on whether the eye is non-glaucomatous or glaucomatous.

However, when the size of the optic nerve head is smaller or larger than the normal size, it is difficult to diagnose glaucoma by the MRA. Based on the Tajimi study, the diagnostic power of detecting glaucoma by the MRA program is reduced when the disc size becomes larger than the normal size [14].

In addition to the MRA, several statistical methods have been proposed by Mikelberg et al., Lester et al. and Bathija et al. to differentiate normal from glaucomatous optic discs [15–17]. Ford et al. reported that these methods of analysis had similar sensitivities once their specificities were equalized. However, the diagnostic power of these methods decreases when the disc size is large, because these methods are based mainly on the data of eyes with a normal disc size [18].

The purpose of this study was to determine the stereometric parameters of eyes with large optic discs without visual field defects. The parameters were compared to those of diagnosed glaucomatous eyes with large optic discs, and we identified the disc parameters which can be used to diagnose glaucoma. We then created a new formula based on these parameters to diagnose glaucoma in eyes with a large optic disc.

## Materials and Methods

### Patients

Patients who were being treated at Shibata Clinic of Ophthalmology from November 2005 to July 2007 were studied. Consecutive subjects who were identified with a large optic disc area >2.82 mm^2^ by the HRT II parameters during the study period underwent visual field examinations with the Humphrey Field Analyzer (HFA) 30-2 SITA Standard program (Carl Zeiss Meditec Inc, Dublin, CA). All HRT tests were performed within six months of the visual field tests.

All participants underwent a comprehensive ophthalmic examination, including a medical and family history, best-corrected visual acuity (BCVA), slit-lamp biomicroscopy, Goldmann applanation tonometry, gonioscopy and fundus examinations. Patients were excluded if they had other eye diseases that could affect the visual field other than glaucoma, those with a BCVA of 20/33 or worse and patients with a tilted optic disc.

### Ethics Statement

All the data were analyzed in the Department of Ophthalmology, Hiroshima University. The study was approved by the Ethics Committee of Hiroshima University (registration number; 957) and was conducted according to the tenets of the Declaration of Helsinki.

In some kind of epidemiological researches like this study design, the Ethics Review Board of the Hiroshima University waived the need for written informed consent from the participants. We didn’t get the written or verbal consent in the medical records. However, we announced the details of this study to the participants using the poster, and have disclosed the results of the examinations and study data to all participants. The Ethics committee of the Hiroshima University had approved the study procedures.

### Evaluation of Visual Fields

A diagnosis of glaucoma was made based on the visual field results obtained with HFA. The results of the HFA 30-2 SITA Standard program were examined by two glaucoma specialists (SO and YK) who were blinded to other information about the eye being examined. The evaluations were carried out on all eyes, excluding those that were unreliable (fixation loss, <20%; false-positive and false-negative, <15%). Abnormal visual field data were defined by the presence of at least one abnormal hemifield that was based on the criteria proposed by Anderson and Patella [19]. The hemifield was judged to be abnormal when the pattern deviation probability plot showed a cluster of three or more non-edge contiguous points having a sensitivity of less than 5% in the upper or lower hemifield, and in one of these with a probability of less than 1%. Glaucomatous visual field changes were diagnosed when the pattern standard deviation was less than 5%, or when the glaucoma hemifield test was abnormal.

### Heidelberg Retinal Tomograph II (HRT II) Parameter Measurements

The optic disc parameters were determined from the HRT II images [11,15,20]. The fundus, including the optic disc, was photographed using the HRT after pupil dilation. The image quality of all the HRT images was checked manually by an experienced operator, and the optic disc margin (contour line) was outlined around the inner margin of the peripapillary scleral rings by the same operator (KY). The analysis was restricted to eyes that had valid optic disc measurements with the HRT II. Good image quality was defined as appropriate focus, brightness and clarity, minimal eye movements, the optic disc centered in the image and a standard deviation of the mean topographic image <40 μm. Eyes in which good-quality images could not be obtained were excluded from the analysis. The results of the MRA were evaluated by classifying borderline cases as being outside the normal limits. The HRT image recording and visual field testing were performed on the same day. All data obtained from the HRT II were analyzed (provide as supplemental data). The RNFL cross sectional area was calculated as the average distance between the retinal surface and a standard point along a contour line of the disc × the length of contour line. The height variation contour (HVC) is the difference in retinal surface height along the contour line of the disc between the highest and lowest points. The cup shape measure represents the overall shape of the optic nerve head and has been shown to have a significant correlation with glaucomatous damage [20,21]. The cup shape measurement is independent of the reference plane and, thus, is unaffected by any variability in the reference plane [22,23]. The reference height is calculated as the average heights of the retina around the disc.

### Statistical Analyses

The eyes with normal visual fields (Non-glaucoma group) and the eyes with a glaucomatous visual field (Glaucoma group) were compared based on the results of the HFA. The glaucoma group was divided into two subsets: an early glaucoma group (MD > −5 dB) and the advanced glaucoma group (MD ≤ −5dB). The correlations between the disc area and each of the HRT parameters were examined in a scatter diagram. Important HRT parameters were selected by using the stepwise method, and the odds ratio for the diagnosis of glaucoma by a logistic regression analysis was calculated. The results of the multiple logistic analyses were used to compare the results of the MRA for the global area. The optimal cut-off value of the receiver operating characteristic curve (ROC) was calculated to differentiate glaucomatous and non-glaucomatous eyes.

Comparisons of the mean values among the groups were made using *t*-tests. The JMP 9.0 Statistical Analysis System software program (SAS Institute Inc. California) was used for the calculations and statistical analyses, and a *P* value <0.05 was considered to be statistically significant.

## Results

### Participants

The demographics of the patients are summarized in Table 1. There were 225 eyes in 145 patients that were analyzed (78 eyes of 49 males and 147 eyes of 96 females). The Non-glaucomatous group included 134 eyes and the glaucoma group included 91 eyes.

**Table 1:** Demographics of the study subjects.

	N	G	*P* *	Glaucoma			
	(n = 134)	(n = 91)		EG	*P* †	AG	*P* ‡
				(n = 66)		(n = 25)	
Age (y.o)	56.70±15.91	63.59±15.20	0.001	62.55±16.50	0.017	66.36±10.85	0.004
SE (D)	−1.27±3.13	−1.60±3.27	0.456	−1.10±2.79	0.713	−2.91±4.06	0.019
MD (dB)	−0.74±1.34	−5.05±5.78	<0.001	−2.43±1.34	<0.001	−11.98±7.32	<0.001
PSD (dB)	1.79±0.88	5.71±4.35	<0.001	3.71±2.40	<0.001	10.97±3.97	<0.001

The data are expressed as the means ± SD.

N: non-glaucomatous group

G: glaucomatous group

EG: early stage glaucoma group (MD <-5dB)

AG: advanced stage glaucoma group (MD ≥-5dB)

SE: spherical equivalent

MD: mean deviation

PSD: pattern standard deviation

* Difference between the normal group and all glaucoma cases

† Difference between the normal group and the early glaucoma group

‡ Difference between the normal group and the progressive/advanced glaucoma group

### Comparison of the Disc Parameters in Non-glaucomatous and Glaucomatous Eyes

The disc parameters were compared between the non-glaucomatous group and the glaucomatous group (Table 2). The disc area in the non-glaucomatous group (3.44 ± 0.50 mm^2^) was not significantly different from that in the glaucomatous group (3.40 ± 0.52 mm^2^). The values of the cup area, cup volume, cup-to-disc area ratio, linear cup/disc ratio, mean cup depth and the maximum cup depth of the glaucomatous group were significantly larger than those of the non-glaucomatous group. The rim area, rim volume, cup shape measure, mean RNFL thickness and RFNL cross-sectional area of the RNFL in the glaucomatous group were significantly smaller than those in the non-glaucomatous group.

**Table 2:** The HRT II parameters in the non-glaucomatous and glaucomatous eyes.

	N	G	*P* *	Glaucoma				
	(n = 134)	(n = 91)		EG	*P* †	AG	*P* ‡	*P* **
				(n = 66)		(n = 25)		
Disc area (mm^2^)	3.44±0.50	3.40±0.52	0.610	3.44±0.56	0.970	3.30±0.37	0.200	0.254
Rim area (mm^2^)	1.96±0.43	1.57±0.43	<0.001	1.68±0.38	<0.001	1.28±0.40	<0.001	<0.001
Rim volume (mm^3^)	0.45±0.19	0.32±0.17	<0.001	0.35±0.16	<0.001	0.25±0.15	<0.001	0.007
Cup area (mm^2^)	1.47±0.47	1.83±0.60	<0.001	1.76±0.63	<0.001	2.02±0.46	<0.001	0.063
Cup volume (mm^3^)	0.37±0.24	0.50±0.26	<0.001	0.49±0.27	0.002	0.53±0.23	0.002	0.512
C/D area	0.42±0.11	0.53±0.13	<0.001	0.51±0.12	<0.001	0.61±0.12	<0.001	<0.001
C/D linear	0.65±0.088	0.73±0.089	<0.001	0.71±0.085	<0.001	0.78±0.079	<0.001	<0.001
Mean CD (mm)	0.29±0.081	0.33±0.086	0.005	0.32±0.084	0.015	0.33±0.091	0.043	0.756
Maximum CD (mm)	0.70±0.16	0.75±0.16	0.025	0.76±0.16	0.017	0.72±0.17	0.450	0.416
CSM	−0.11±0.059	−0.085±0.062	0.001	−0.098±0.059	0.110	−0.052±0.059	<0.001	0.001
RNFLT (mm)	0.23±0.077	0.19±0.073	<0.001	0.20±0.073	0.006	0.17±0.072	<0.001	0.097
RNFL CS (mm^2^)	1.48±0.51	1.23±0.50	<0.001	1.29±0.51	0.011	1.08±0.46	<0.001	0.075
HVC (mm)	0.38±0.11	0.38±0.13	0.830	0.39±0.12	0.500	0.36±0.15	0.490	0.345
RH (mm)	0.38±0.10	0.36±0.10	0.300	0.36±0.10	0.170	0.38±0.11	0.910	0.329

The data are expressed as the means ± SD.

N: non-glaucomatous group

G: glaucomatous group

EG: early stage glaucoma group (MD<-5dB)

AG: advanced stage glaucoma group (MD ≥-5dB)

C/D area: cup-to-disc area ratio

C/D linear: linear cup/disc ratio

CD: cup depth

CSM: cup shape measure

RNFLT: mean RNFL thickness

RNFL CS: RNFL cross-sectional area

HVC: height variation contour

RH: reference height

* Difference between the normal patients and all glaucoma cases

† Difference between the normal and early glaucoma groups

‡ Difference between the normal and progressive/advanced glaucoma groups

** Difference between the early glaucoma and progressive/advanced glaucoma groups

### Correlations between the Disc Area and the HRT Parameters

**Fig 1:**
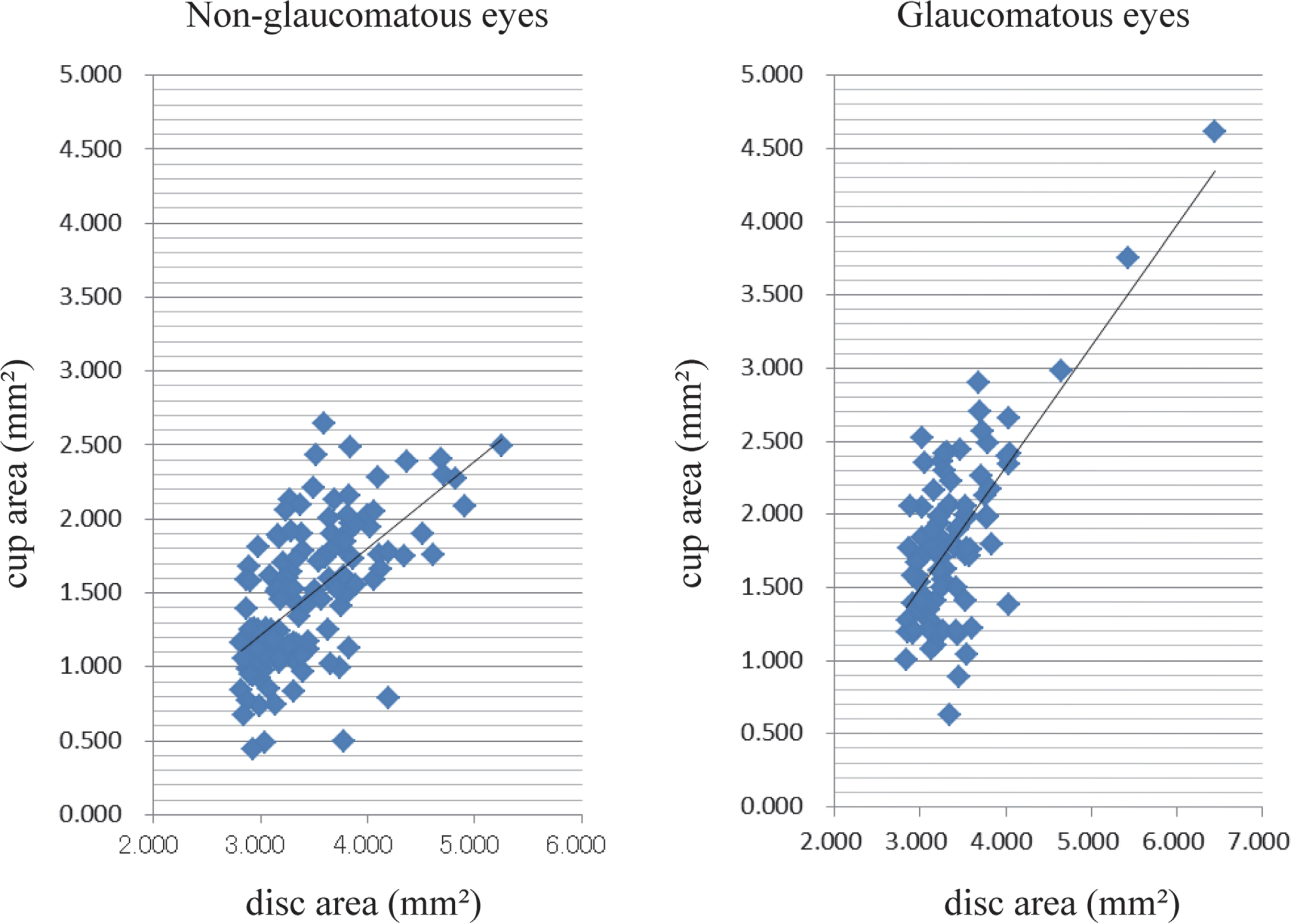
Scatter diagrams showing the correlations between the disc area and cup area. (Left figure) R^2^ = 0.384, cup area = 0.585 x disc area—0.540. (Right figure) R^2^ = 0.515, cup area = 0.825 x disc area—0.974.

A scatter diagram shows that the cup area increased with an increase of the disc area in both the non-glaucomatous and glaucomatous groups (Fig. 1). No other HRT parameter was significantly correlated with the disc area (data not shown).

### The Sensitivity and Specificity of Moorfield’s Regression Analysis (MRA)

The sensitivity of the MRA was 59.0% and the specificity was 66.0% for the present patients (Table 3). The sensitivity decreased when the subjects with advanced glaucoma were excluded (Table 4). Otherwise, the sensitivity improved when the subjects in the early glaucoma group were excluded (Table 5).

**Table 3:** The sensitivity and specificity of the MRA (Moorfield’s Regression Analysis) for all subjects (n = 225).

	MRA	Sector						
	Classification	global	temporal	tmp/sup	tmp/inf	nasal	nsl/sup	nsl/inf
Sensitivity (%)	88	59	34	39	65	54	54	66
Specificity (%)	36	66	88	90	66	57	70	61

tmp: temporal

nsl: nasal

sup: superior

inf: inferior

**Table 4:** The sensitivity and specificity of the MRA (Moorfield’s Regression Analysis) for the non-glaucomatous group and the early stage glaucoma group (n = 200).

	MRA	Sector						
	Classification	global	temporal	tmp/sup	tmp/inf	nasal	nsl/sup	nsl/inf
Sensitivity (%)	86	51	25	29	59	48	48	58
Specificity (%)	36	66	88	90	66	57	70	61

tmp: temporal

nsl: nasal

sup: superior

inf: inferior

**Table 5:** The sensitivity and specificity of the MRA (Moorfield’s Regression Analysis) for the non-glaucomatous group and the advanced stage glaucoma group (n = 159).

	MRA	Sector						
	Classification	global	temporal	tmp/sup	tmp/inf	nasal	nsl/sup	nsl/inf
Sensitivity (%)	96	80	60	68	80	72	72	88
Specificity (%)	36	66	88	90	66	57	70	61

tmp: temporal

nsl: nasal

sup: superior

inf: inferior

### The Stepwise Logistic Regression Analysis

**Fig 2:**
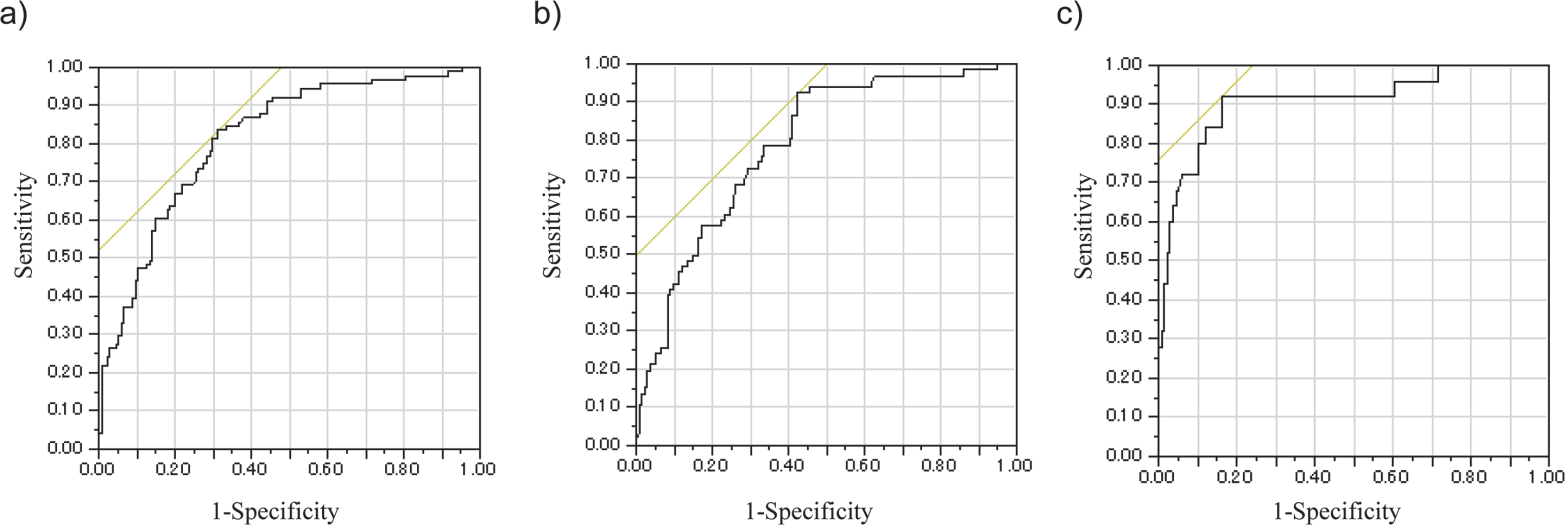
The ROC curves of the logistic regression analyses with the stepwise method in which glaucoma was the outcome variable among all subjects.

A stepwise logistic regression analysis showed that an increase of the cup-to-disc area ratio, an increase of the height variation contour (HVC) and a decrease of the RNFL cross-sectional area were risk factors for glaucoma in eyes with a large optic disc (Table 6). The formula generated by the logistic regression analysis showed that the area under the ROC was 0.81. The sensitivity and specificity of diagnosing glaucoma were 83.5% and 68.7%, respectively, if the cut off value used for the classification was −0.69 (Fig. 2A).

**Table 6:** The results of the logistic regression analysis with a stepwise method, in which glaucoma was the outcome variable among all subjects.

Variable	Odds Ratio	95% Confidence Interval			*P*
Cup volume (×10^−1^)	0.83	0.67	-	1.03	0.10
C/D area (×10^−1^)	2.72	1.70	-	4.52	<.0001
HVC (×10^−1^)	3.14	1.80	-	5.81	0.0001
RNFL CS	0.085	0.021	-	0.31	0.0003

The yellow line is a straight line, with an angle of 45 degrees which touches the ROC curve. a) The analysis for all subjects; b) The analysis for the non-glaucomatous and early stage glaucoma groups; c) The analysis for the non-glaucomatous and advanced stage glaucoma groups.

Before stepwise selection, there were 13 factors (disc area, cup area, cup-to-disc area ratio, rim area, height variation contour, cup volume, rim volume, mean cup depth, maximum cup depth, cup shape measure, mean RNFL thickness, RNFL cross-sectional area and reference height). The four factors shown in the table were selected using a stepwise method.

The formula generated by the logistic regression: F = −5.26 + (−1.80) x Cup volume + 9.99 x C/D area + 11.44 x HVC + (−2.461) x RNFL CS.

R^2^ = 0.22, Significance of the model: *p*<0.0001

The analysis of the subjects in the Non-glaucomatous group and early glaucoma group by the logistic regression analysis with the stepwise method, in which glaucoma was the outcome variable, showed that an increase of the cup-to-disc area ratio, an increase of the HVC and a decrease of the RNFL cross-sectional area were risk factors for early stage glaucoma (Table 7). The formula generated by the logistic regression analysis showed that the area under the ROC was 0.79. The sensitivity and specificity for diagnosing glaucoma were 92.4% and 57.5%, respectively, if the cut-off value used for the classification was −1.19 (Fig. 2B).

**Table 7:** The results of the logistic regression analysis with the stepwise method, in which glaucoma was the outcome variable between the non-glaucomatous group and the early stage glaucoma group.

Variable	Odds Ratio	95% Confidence Interval			*P*
C/D area (×10^–1^)	1.71	1.25	-	2.38	0.001
HVC (×10^–1^)	3.42	1.93	-	6.45	<.0001
RNFL CS	0.073	0.018	-	0.27	<.0001

Before the stepwise selection, there were 13 factors (shown in Table 6). The three factors shown in the table were selected using with a stepwise method.

The formula generated by the logistic regression: F = −4.18 + 5.35 x C/D area + 12.3 x HVC + (−2.6) x RNFL CS.

C/D area: cup-to-disc area ratio; HVC: height variation contour;

RNFL CS: RNFL cross-sectional area

R^2^ = 0.16, Significance of the model: *p*<0.0001

An analysis of the subjects in the non-glaucomatous group and the advanced glaucoma group by a stepwise logistic regression analysis showed that an increase in the cup-to-disc area ratio and a decrease of the cup volume were risk factors for glaucoma (Table 8). The formula generated by the logistic regression analysis showed that the area under the ROC was 0.91. The sensitivity and specificity for diagnosing glaucoma were 92.0% and 83.7%, respectively, if the cut-off value used for the classification was −1.94 (Fig. 2C).

**Table 8:** The results of the logistic regression analysis with the stepwise method, in which glaucoma was the outcome variable between the non-glaucomatous group and the advanced stage glaucoma group.

Variable	Odds Ratio	95% Confidence Interval			*P*
Cup volume (×10^−1^)	0.54	0.35	-	0.77	0.002
C/D area (×10^−1^)	11.96	5.23	-	33.77	<0.001

Before stepwise selection, there were 13 factors (shown in Table 6). The two factors shown in the table were selected with a stepwise method.

The formula generated by the logistic regression: F = −11.66 + (−6.17) x Cup volume + 24.81 x C/D area.

C/D area: cup-to-disc area ratio

R^2^ = 0.44, Significance of the model: *p*<0.0001

## Discussion

It is difficult to recognize the morphological changes in a glaucomatous optic disc when the disc size is large because a large disc area is significantly correlated with the optic cup area in both glaucomatous and non-glaucomatous eyes. We therefore determined the morphological features of large optic discs in eyes that were diagnosed with glaucoma by HFA. We were also able to develop a new formula to diagnose glaucoma based on the stereometric parameters in eyes with large optic discs.

### The Differences in the Morphological Features of Large Discs Between Glaucomatous and Non-glaucomatous Eyes

Our results showed that all of the parameters related to disc cupping, viz., the cup area, cup volume, cup-to-disc area ratio, linear cup/disc ratio, mean cup disc and maximum cup depth, were significantly larger in the eyes with glaucoma than in the eyes without glaucoma. The parameters related to the disc rim, viz., the rim area, rim volume and the circumpapillary nerve fiber layer thickness (mean RNFL thickness, RNFL cross-sectional area) in glaucomatous eyes were significantly smaller than those in eyes without glaucoma.

Similar findings were previously noted in eyes with normal-sized optic discs. For example, Medeiros reported that the rim-related parameters, including the rim area, rim volume and rim-to-disc area, of glaucomatous eyes were significantly smaller than those in eyes without glaucoma [24]. Medeiros also reported statistically significant differences in the cup parameters, viz., the cup-to-disc area ratio and the linear cup-to-disc ratio, between glaucomatous and non-glaucomatous eyes with normal-sized optic discs [24]. Uchida et al. reported that the area and volume of the optic cup were significantly larger in glaucomatous eyes than in normal eyes [20].

Our results on eyes with large optic discs showed that the maximum cup depth and mean cup depth in the early stage glaucoma group was significantly larger than those in the non-glaucomatous group (Table 2). However, the differences in these parameters between the early stage and advanced stage glaucoma groups were small, and the differences were not significant. Thus, the evaluation of whether glaucoma has progressed by using the cup depth parameters does not lead to accurate results. On the other hand, the rim area in advanced glaucomatous eyes was significantly smaller than that in the early stage glaucomatous subjects, and the cup-to-disc area ratio and linear cup/disc ratio were significantly larger in advanced stage than in early stage glaucomatous eyes. These results indicate that the cup depth reaches its maximum change at the early stage of glaucoma, but that the rim thinning continues to decrease for a longer duration in eyes with large optic discs.

### Diagnosing Glaucoma by the HRT Stereometric Parameters in Eyes with Large Optic Discs

We used the stereometric parameters determined by HRT II to create a new formula for eyes with a large optic disc. We used the MRA to determine the validity of the new formula, because it is the default program included for the HRT II and is easy to use. The MRA uses the disc rim area, disc area and age for its calculations. In our study, an increase in the cup-to-disc area ratio, an increase in the HVC and a reduction of the RNFL cross-sectional area were risk factors for glaucoma. The HVC is the difference in the height between the highest retinal surface and the lowest retinal surface along the contour line. The RNFL cross-sectional area is the average distance between the retinal surface and a standard point along the contour line of the disc x the length of contour line. Both parameters are related to the thickness of the circumpapillary retinal nerve fiber layer. An increase of the HVC and decrease of the RNFL cross-sectional area suggest glaucomatous changes, because the RNFL becomes thinner in glaucomatous eyes. The diagnostic power, which is the sum of the sensitivity and specificity, of our new formula that included the cup volume, cup-to-disc area ratio, HVC and RNFL cross-sectional area was 1.53, whereas the diagnostic power of the MRA was 1.25. The large discs are identical to normal-sized discs in terms of the RNFL thickness [10,25]. Oddone et al. showed that the diagnostic accuracy of quantitative RNFL assessments measured with Cirrus HD-OCT and GDx-VCC is high and insignificantly affected by the size of the optic disc [26]. Furthermore, they reported that it may provide more consistent diagnostic outcomes across small and large discs when compared with the optic nerve head assessment, such as the MRA program measured with HRT [26]. Therefore, it is important to evaluate the parameters related to the retinal nerve fiber thickness, which are not affected by the disc size, to diagnose glaucoma in eyes with large optic discs.

The parameters related to the RNFL thickness were not selected by a logistic regression analysis when we differentiated eyes in the advanced stage and non-glaucomatous groups. These findings are not contradictory to the study by Hood et al., who reported a good correlation between the circumpapillary RNFL thickness obtained by OCT and the visual field loss (Mean Deviation, MD) when the field loss was >−6 dB. This is because when the MD is < −6.0 dB, the RNFL changes are too small to detect the progression of glaucoma [27].

Another interesting result of this study was the high incidence, 40.4%, of glaucoma in eyes with a large optic disc. In the general population, the incidence of glaucoma was 5.0% in the Tajimi study and 3.5% in the Namil study [28,29]. It is difficult to draw conclusions about the prevalence of glaucoma in cases with large discs, because this was a single center case-control study. However, all subjects with a disc > 2.82mm^2^ by HRT II were included in this study. This suggested that a large optic disc may be a risk factor for glaucoma. Our present findings are similar to past reports suggesting that a large optic disc is a risk factor for glaucoma [30–32].

There are a few limitations associated with our study. First, this was a single center, case-control study, and we analyzed both eyes of each patient when the subjects had large discs in both eyes. Abe et al. reported that all HRT parameters were significantly correlated between right and left eyes. However, their results suggested that subjects with larger discs tended to show greater inter-eye absolute differences in these HRT parameters [33]. In our 147 consecutive cases, 69 subjects had a large disc in one eye and 78 subjects had a large disc in both eyes. The disc features were not correlated between the right and left eyes. This is the reason why we included the data from both eyes even when both eyes had a large disc. Second, we have not confirmed the ability of our new formula to discriminate the glaucoma group from the non-glaucoma group in another set of subjects with a large optic disc area.

In conclusion, our data indicate that the cup-to-disc area ratio, HVC and RNFL cross-sectional area are significant HRT parameters that are more appropriate for diagnosing early stage glaucoma, and an increase in the cup-to-disc area ratio and a decrease of the cup volume were risk factors for advanced glaucoma in eyes with large optic discs.

## Supporting Information

S1 Dataset The patients’ information, HFA, and HRT data. (XLS)
